# TLR4-interactor with leucine-rich repeats (TRIL) is involved in diet-induced hypothalamic inflammation

**DOI:** 10.1038/s41598-021-97291-7

**Published:** 2021-09-09

**Authors:** Alexandre Moura-Assis, Pedro A. S. Nogueira, Jose C. de-Lima-Junior, Fernando M. Simabuco, Joana M. Gaspar, Jose Donato Jr, Licio A. Velloso

**Affiliations:** 1 Laboratory of Cell Signalling-Obesity and Comorbidities Research Center, University of Campinas, Campinas, Brazil; 2 Multidisciplinary Laboratory of Food and Health (LABMAS), School of Applied Sciences (FCA), University of Campinas (UNICAMP), Limeira, São Paulo Brazil; 3 Department of Physiology and Biophysics, Institute of Biomedical Sciences, University of Sao Paulo, Sao Paulo, Brazil; 4 National Institute of Science and Technology on Neuroimmunomodulation, Rio de Janeiro, Brazil

**Keywords:** Feeding behaviour, Metabolic diseases

## Abstract

Obesity and high-fat diet (HFD) consumption result in hypothalamic inflammation and metabolic dysfunction. While the TLR4 activation by dietary fats is a well-characterized pathway involved in the neuronal and glial inflammation, the role of its accessory proteins in diet-induced hypothalamic inflammation remains unknown. Here, we demonstrate that the knockdown of TLR4-interactor with leucine-rich repeats (Tril), a functional component of TLR4, resulted in reduced hypothalamic inflammation, increased whole-body energy expenditure, improved the systemic glucose tolerance and protection from diet-induced obesity. The POMC-specific knockdown of Tril resulted in decreased body fat, decreased white adipose tissue inflammation and a trend toward increased leptin signaling in POMC neurons. Thus, Tril was identified as a new component of the complex mechanisms that promote hypothalamic dysfunction in experimental obesity and its inhibition in the hypothalamus may represent a novel target for obesity treatment.

## Introduction

The hypothalamus is a key brain region involved in the regulation of food intake and systemic homeostasis. In diet-induced obesity (DIO), a chronic and low-grade proinflammatory response in the arcuate nucleus (ARC) of the hypothalamus impairs the neuronal control of energy balance^1,2^. Thus, to understand how such inflammation develops in the hypothalamus is pivotal in determining obesity treatment.

Saturated fatty acids from hypercaloric diets can serve as ligands for the toll-like receptor-4 (TLR4)^3^ and genetic mouse models lacking either TLR4^4^ or its downstream accessory protein myeloid differentiation primary response gene 88 (MyD88)^5^ are protected from DIO and hypothalamic inflammation. Despite progress towards characterization of TLR4 signaling, the role of other critical components of TLR4 signaling in the hypothalamus remain poorly understood.

The TLR4-interactor with leucine-rich repeats (Tril) is a single transmembrane spanning 89 kDa protein that contains 13 leucine-rich repeats and is highly expressed in the brain^6^. It plays an important role mediating TLR4 signal transduction, and whole-body Tril knockout results in defective inflammatory cytokine production in response to LPS and *E. coli*, particularly in the central nervous system (CNS)^7^. A previous study has shown an enrichment of Tril in proopiomelanocortin-expressing (POMC) neurons^8^, a leptin-responsive and appetite-suppressant group of hypothalamic neurons largely affected by obesity^9–11^. Here, we hypothesized that Tril might be involved in diet-induced hypothalamic inflammation and in the regulation of POMC neurons. We show that knockdown of hypothalamic Tril protects mice from diet-induced body mass gain and systemic glucose intolerance.

## Results

### Hypothalamic Tril expression is increased in diet-induced obesity

**Figure 1:**
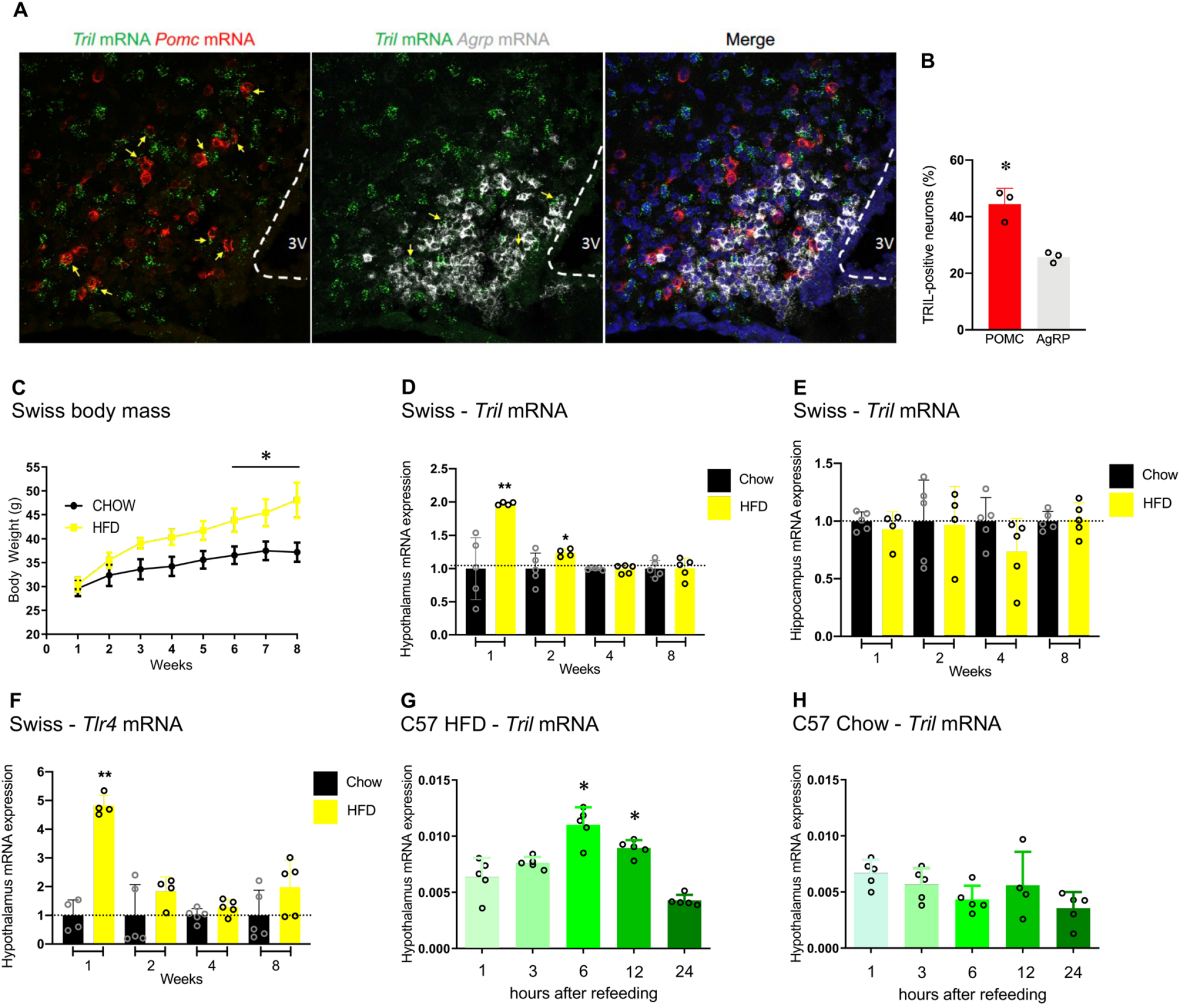
The impact of a high-fat diet on the expression of Tril. The hypothalamic Tril mRNA was analyzed in POMC and AgRP neurons using fluorescent in situ hybridization (**A**) and the overlap with POMC and AgRP neurons was quantified (**B**). In (**C**–**F**), Swiss mice were fed chow or a high-fat diet (HFD) for 1 to 8 weeks; body mass is shown in **C**; transcript expressions of *Tril* (**D**, hypothalamus; **E**, hippocampus) and *Tlr4* (**F**, hypothalamus) in mice fed a HFD are plotted as normalized for the respective expressions in mice fed chow. C57BL/6J mice fed a HFD (**G**) or chow (**H**) were submitted to an overnight fast and hypothalami were extracted after 1, 6, 12 or 24 h of refeeding for determination of *Tril* mRNA expression. In A, images are representative of three independent samples and. In (**C**–**F**), n = 4–5; *p < 0.05 and **p < 0.01 vs. respective control (chow). In (**G**, **H**), n = 5; *p < 0.05 vs. 1 h. Two-tailed Student’s *t*-test or two-way analysis of variance (ANOVA) followed by Bonferroni post-hoc test were used.

*Tril* mRNA expression was detected in 44% of POMC neurons and only 25% of AgRP neurons (Fig. 1A,B); the major Tril overlap with POMC neurons was further confirmed in hypothalamic slices from POMC-GFP mice (Suppl. Fig. 1A,D) in comparison with AgRP-tdTomato mice (Suppl. Fig. 1B). Tril was also detected in Iba1-expressing glial cells (Suppl. Fig. 1C). In outbred obesity-prone Swiss mice (Fig. 1C), the hypothalamic (Fig. 1D) but not hippocampal (Fig. 1E) expression of *Tril* underwent increase one and two weeks after the introduction of HFD. This was accompanied, in the first week, by the increased expression of hypothalamic *Tlr4* (Fig. 1F). In isogenic C57BL/6J mice, the hypothalamic expression of Tril was increased after refeeding in mice fed a HFD (Fig. 1G) but not in mice fed a standard chow (Fig. 1H).

### Inhibition of hypothalamic Tril protects from diet-induced obesity

**Figure 2:**
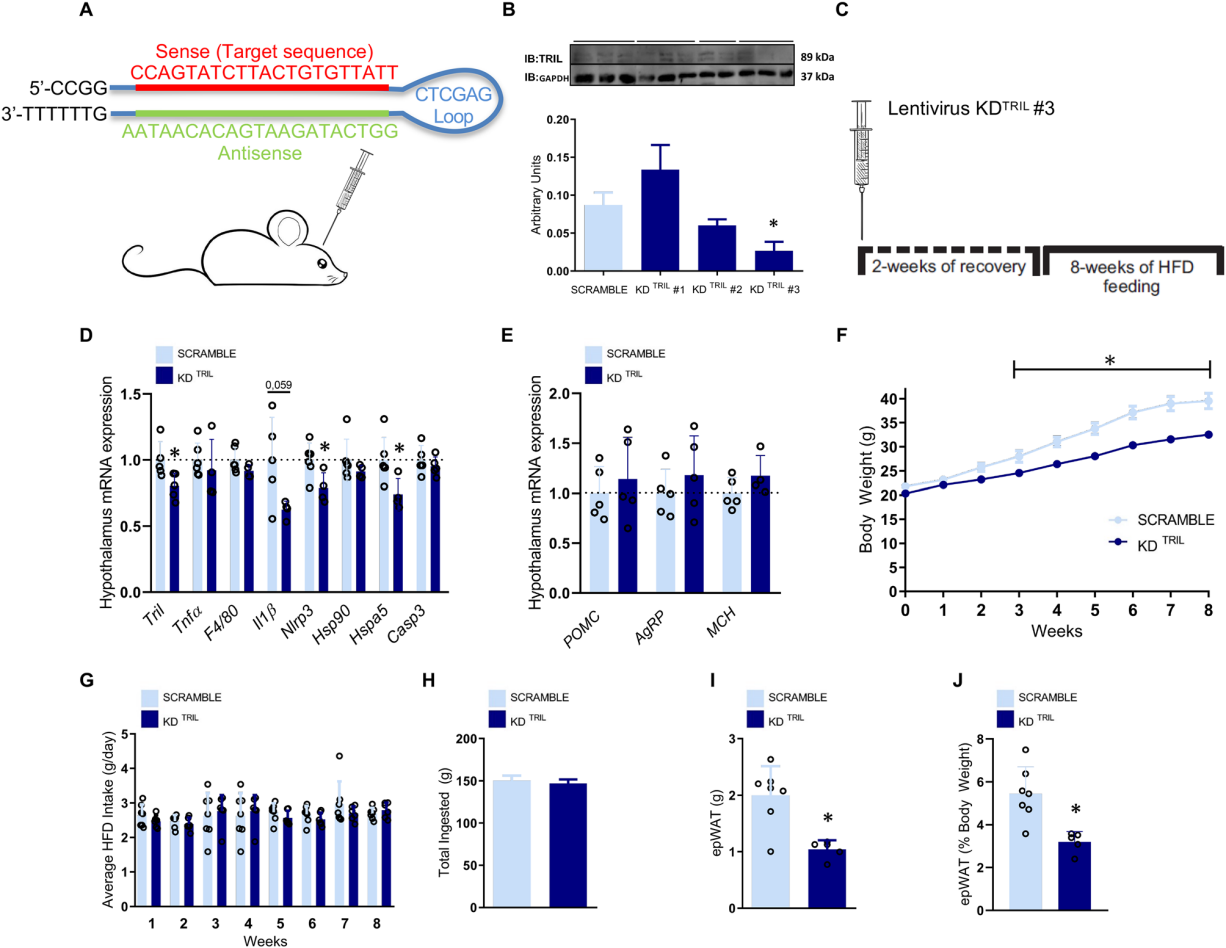
The inhibition of hypothalamic Tril affects body mass and adiposity. C57BL/6J mice were submitted to an injection into the arcuate nucleus of a lentivirus carrying a scramble or one out of three different shRNA sequences for targeting Tril (KD^TRIL^#1, KD^TRIL^#2 or KD^TRIL^#3) (**A**, **B**); immunoblot of hypothalamic extracts was employed to determine Tril expression in each experimental group (**B**); the sequence of the selected inhibitory sequence, KD^TRIL^#3, is depicted in (**A**). The protocol employed in the experiments is depicted in (**C**). The hypothalamic transcript expression of inflammatory and apoptotic genes (D) and neurotransmitter genes (**E**) was determined at the end of the experimental period. Body mass (**F**) and food intake (**G**, **H**) were determined during the experimental period. Total (**I**) and relative (**J**) epididymal fat mass were determined at the end of the experimental period. In (**B**), n = 4; in (**D**–**J**), n = 5–7. In all experiments *p < 0.05 vs. scramble. epWAT, epididymal white adipose tissue; HFD, high-fat diet; KD, knockdown. Two-tailed Student’s *t*-test or two-way analysis of variance (ANOVA) followed by Bonferroni post-hoc test were used.

Three distinct lentiviral clones containing a shRNA to knockdown *Tril* were injected in the ARC and a scramble was used as a negative, non-silencing control (Fig. 2A,B); the lentivirus clone #3 (sequence depicted in Fig. 2A and Tril protein inhibition in Fig. 2B) generated a significant Tril knockdown. The protocol (using lentivirus #3) was designed to evaluate whether the knockdown of hypothalamic Tril could prevent the effect of a HFD on metabolic parameters, since the introduction of a HFD occurred after the injection of the lentivirus particles (Fig. 2C). The inhibition of hypothalamic Tril resulted in the reduction of transcript expression of hypothalamic *Il1ß*, *Nlrp3* and *Hspa*5 (Fig. 2D); no changes were detected in baseline expression of transcripts encoding for *Pomc*, *Agrp* and *Mch* (Fig. 2E). The inhibition of hypothalamic Tril resulted in reduced body mass gain (Fig. 2F) and no change in caloric intake (Fig. 2G and 2H); in addition, inhibition of hypothalamic Tril resulted in reduced absolute (Fig. 2I) and relative (Fig. 2J) epididymal white adipose tissue mass.

### Inhibition of hypothalamic Tril improves systemic glucose tolerance

**Figure 3:**
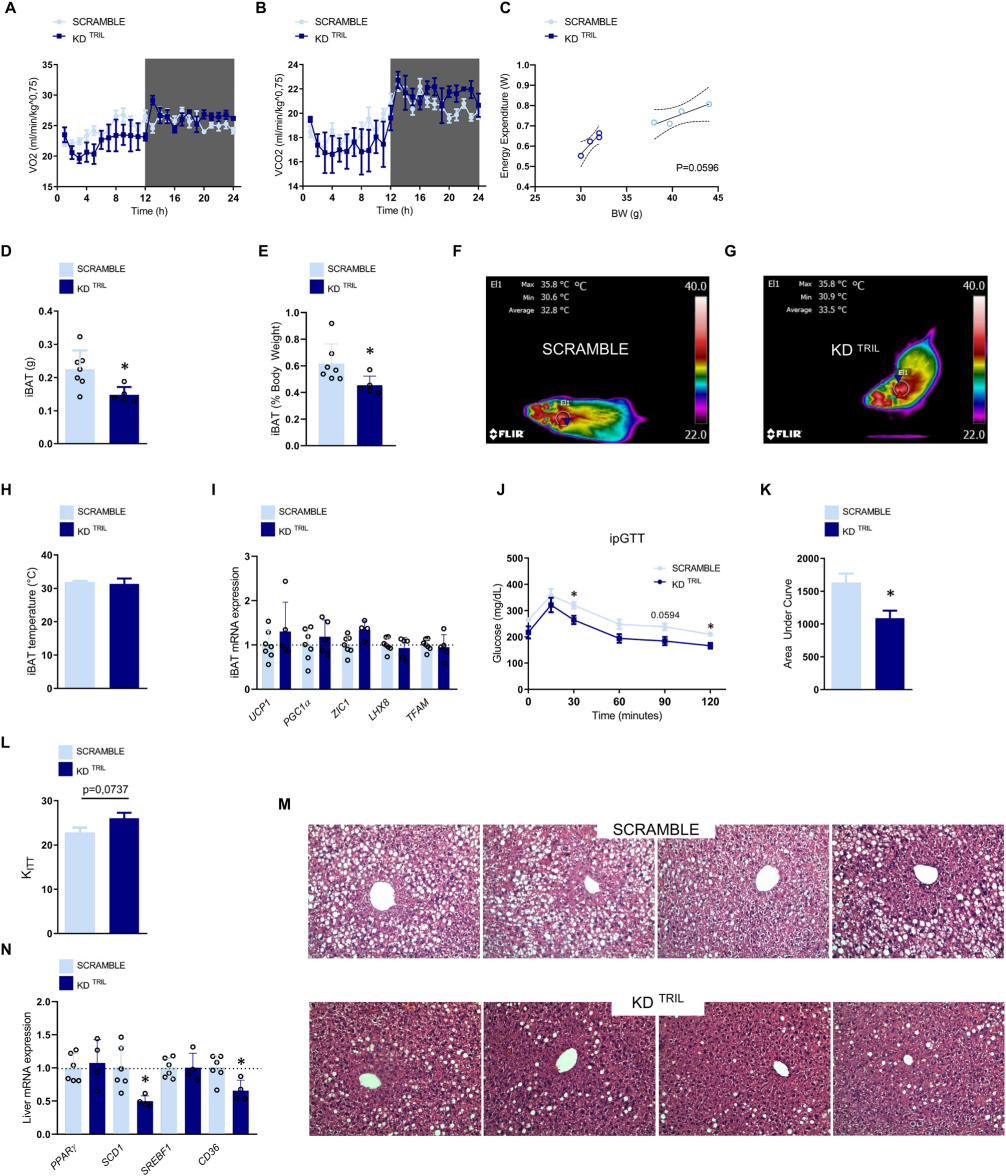
The inhibition of hypothalamic Tril affects energy expenditure and systemic metabolic parameters. C57BL/6J mice were submitted to an injection into the arcuate nucleus of a lentivirus carrying a scramble or a shRNA sequence (sequence KD^TRIL^#3) employing the same experimental protocol as depicted in Fig. 2C. At the end of the experimental period, mice were submitted to determination of O_2_ consumption (**A**), CO_2_ production (**B**), energy expenditure (**C**), determination of interscapular brown adipose tissue total (**D**) and relative (**E**) mass, determination of interscapular temperature (**F**–**H**), determination of interscapular brown adipose transcript expression of thermogenic genes (**I**), determination of whole-body glucose tolerance by means of an intraperitoneal glucose tolerance test (**J**), area under the curve of blood glucose levels (**K**) and determination of whole body insulin sensitivity by means of an insulin tolerance test (**L**, constant of blood glucose decay during the insulin tolerance test). The liver was extracted for histological examination (**M**) and also for determination of transcript expression of genes involved in lipogenesis or lipid uptake (**N**). In (**A**–**C**), n = 4 and in (**D**–**N**), n = 5–7; *p < 0.05 vs. scramble. In M, images are representative of four independent experiments. EE, energy expenditure; iBAT, interscapular brown adipose tissue; ipGTT, intraperitoneal glucose tolerance test; Kitt, constant of blood glucose decay during the insulin tolerance test. Two-tailed Student’s *t*-test or two-way analysis of variance (ANOVA) followed by Bonferroni post-hoc test were used. The energy expenditure was calculated in watts units (W) using a modified Weir equation and the analysis of covariance (ANCOVA) was performed for the analysis of the effect of body weight on energy expenditure.

In mice under hypothalamic knockdown of Tril expression and fed a HFD (as depicted in the experimental protocol in Fig. 2C), there was a trend in the increase of energy expenditure (Fig. 3A–C). The absolute (Fig. 3D) and relative (Fig. 3E) mass of the interscapular brown adipose tissue (iBAT) were reduced in comparison to control, but no changes neither in the temperature (Fig. 3F–H) of iBAT nor in the expression of iBAT transcripts encoding proteins involved in thermogenesis (Fig. 3I) were found. Moreover, the inhibition of hypothalamic Tril improved systemic glucose tolerance (Fig. 3J,K), promoted a trend to increase systemic insulin sensitivity (Fig. 3L) and reduced liver steatosis (Fig. 3M), which was accompanied by reduced hepatic expression of transcripts encoding for *Scd1* and *CD36* (Fig. 3N).

### POMC-specific knockdown of Tril reduces body adiposity and increases hypothalamic responsiveness to leptin

**Figure 4:**
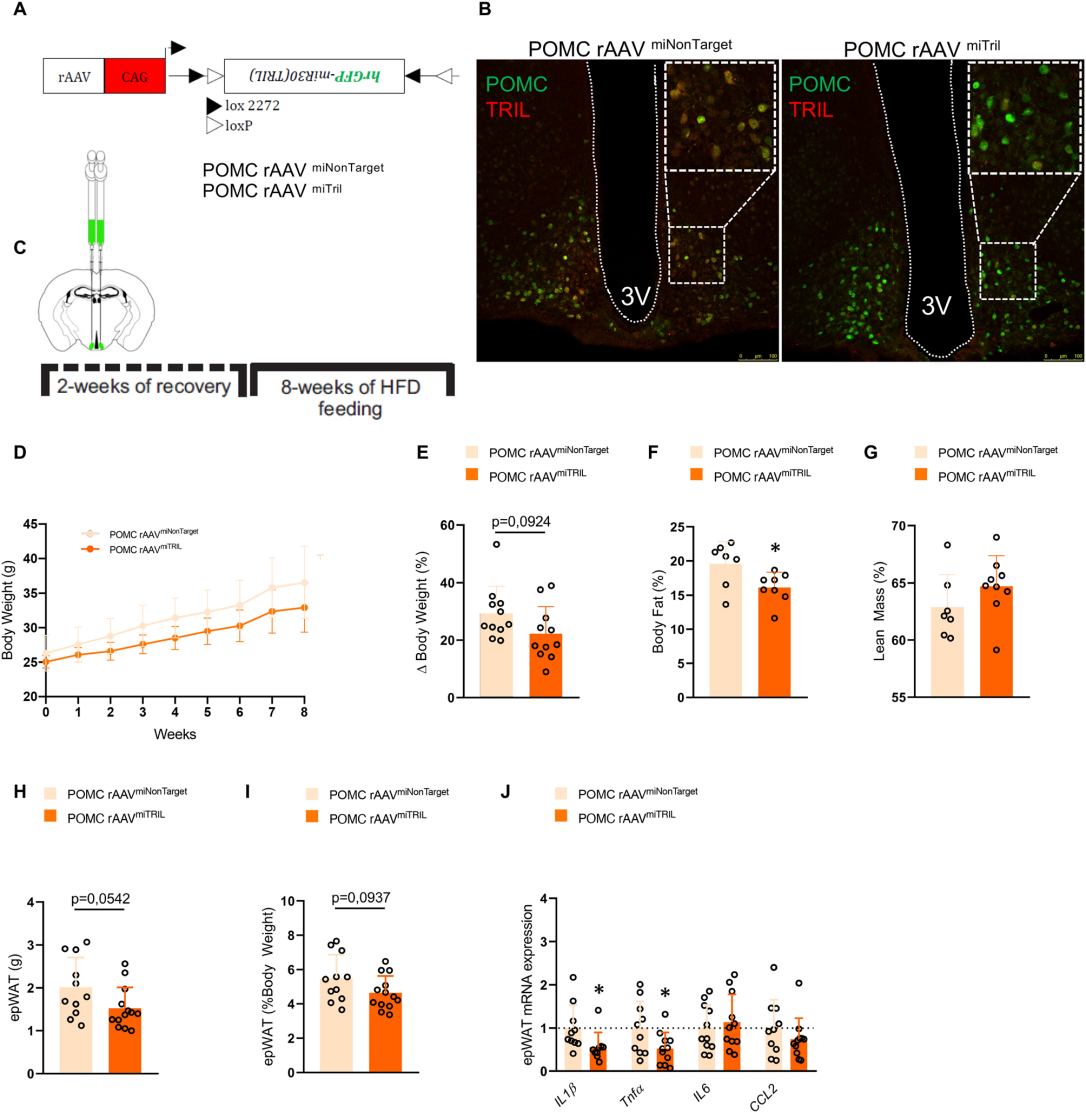
POMC-specific knockdown of Tril reduces body fat. POMC-Cre mice were submitted to an intracerebroventricular injection with Cre-dependent AAV-FLEX-EGFP-mir30 (TRIL) carrying either a non-target sequence (rAAVmiNonTarget) or a Tril targeting sequence (rAAVmiTRIL) (**A**). GFP fluorescence exclusively in the ARC after bilateral infusions of AAV-FLEX-EGFP-mir30 (TRIL) into the hypothalamus of Pomc-Cre mice (**B**). The protocol employed in the experiments is depicted in C. Body mass (**D**, **E**) was determined throughout the experimental period. Relative fat (**F**) and lean (**G**) mass as well as the absolute (**H**) and relative (**I**) epididymal fat mass were determined at the end of the experimental period. The expressions of inflammatory genes were determined in the epididymal adipose tissue at the end of the experimental period (**J**). In (**D**–**E**) and (**H**–**J**), n = 11; in F-G, n = 7–8; *p < 0.05 vs. miNonTarget. Two-tailed Student’s *t*-test or two-way analysis of variance (ANOVA) followed by Bonferroni post-hoc test were used.

**Figure 5:**
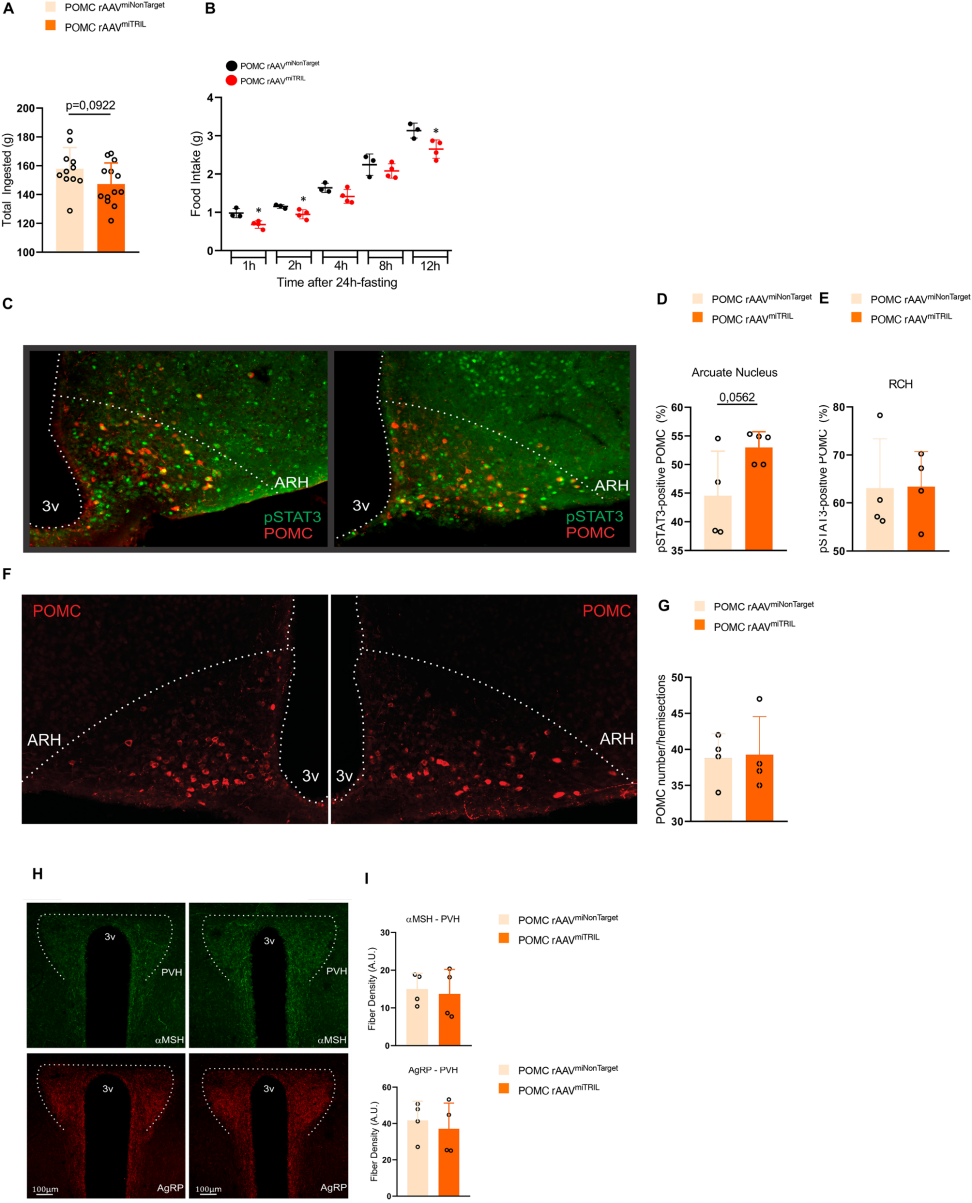
Inhibition of Tril in POMC neurons improves leptin sensitivity. POMC-Cre mice were submitted to an intracerebroventricular injection with Cre-dependent AAV-FLEX-EGFP-mir30 carrying either a non-target sequence (rAAVmiNonTarget) or a Tril targeting sequence (rAAVmiTRIL) and then submitted to the same protocol as in Fig. 4C. The cumulative consumption of diet was determined throughout the experimental period (**A**). At the end of the experimental period, mice were submitted to a determination of spontaneous food intake after a period of 24 h fasting (**B**). Leptin-induced phosphorylation of STAT3 was determined by calculating the proportion of phospho-STAT3 per POMC-positive cells in the arcuate nucleus (**D**) and retrochiasmatic hypothalamus (**E**) employing immunofluorescence staining; an illustrative image obtained from the mediobasal hypothalamus is depicted in (**C**). No difference was observed in the number of POMC neurons in the ARC (**F**, **G**). Immunofluorescence staining was employed to determine the density of α-MSH (**H**, upper panels and **I**) and AgRP (**H**, lower panels and **I**) fiber projections to the paraventricular hypothalamus. In (**B**–**H**), n = 4–5; in (**D**, **E** and **G**), n = 5; *p < 0.05 vs. miNonTarget. Two-tailed Student’s *t*-test was used.

A Cre-dependent AAV was designed to selectively knockdown Tril in POMC neurons of the ARC of POMC-Cre mice (Fig. 4A,B). Since the introduction of a HFD occurred after the injection of rAAV, this protocol was designed to evaluate whether the knockdown of Tril in POMC neurons could prevent the detrimental effects of HFD feeding on metabolic parameters (Fig. 4C). This approach resulted in a trend to overcome HFD-induced body mass gain (Fig. 4D,E) and a significant reduction of fat (Fig. 4F) but not lean mass (Fig. 4G). In addition, there were trends to reduce absolute (Fig. 4H) and relative (Fig. 4I) epididymal fat mass and significant reductions of epididymal adipose tissue expression of the inflammatory transcripts *Il1*β and *Tnfα* (Fig. 4J). The knockdown of Tril in POMC neurons resulted in a trend to reduce cumulative food intake throughout the experimental period (Fig. 5A) and a significant reduction of food intake acutely after a period of prolonged fasting (Fig. 5B). This was accompanied by increased leptin-induced activation of STAT3 in POMC neurons (Fig. 5C,D) but not in the retrochiasmatic hypothalamus (Fig. 5E). Inhibiting Tril in POMC neurons promoted no modification in the density of αMSH (Fig. 5H, upper panels and Fig. 5I) and AgRP (Fig. 5H, lower panels and Fig. 5I) projections to the paraventricular hypothalamus and it did not alter POMC number (Fig. 5F,G).

### POMC-specific knockdown of Tril increases the thermogenic gene expression in brown adipose tissue

**Figure 6:**
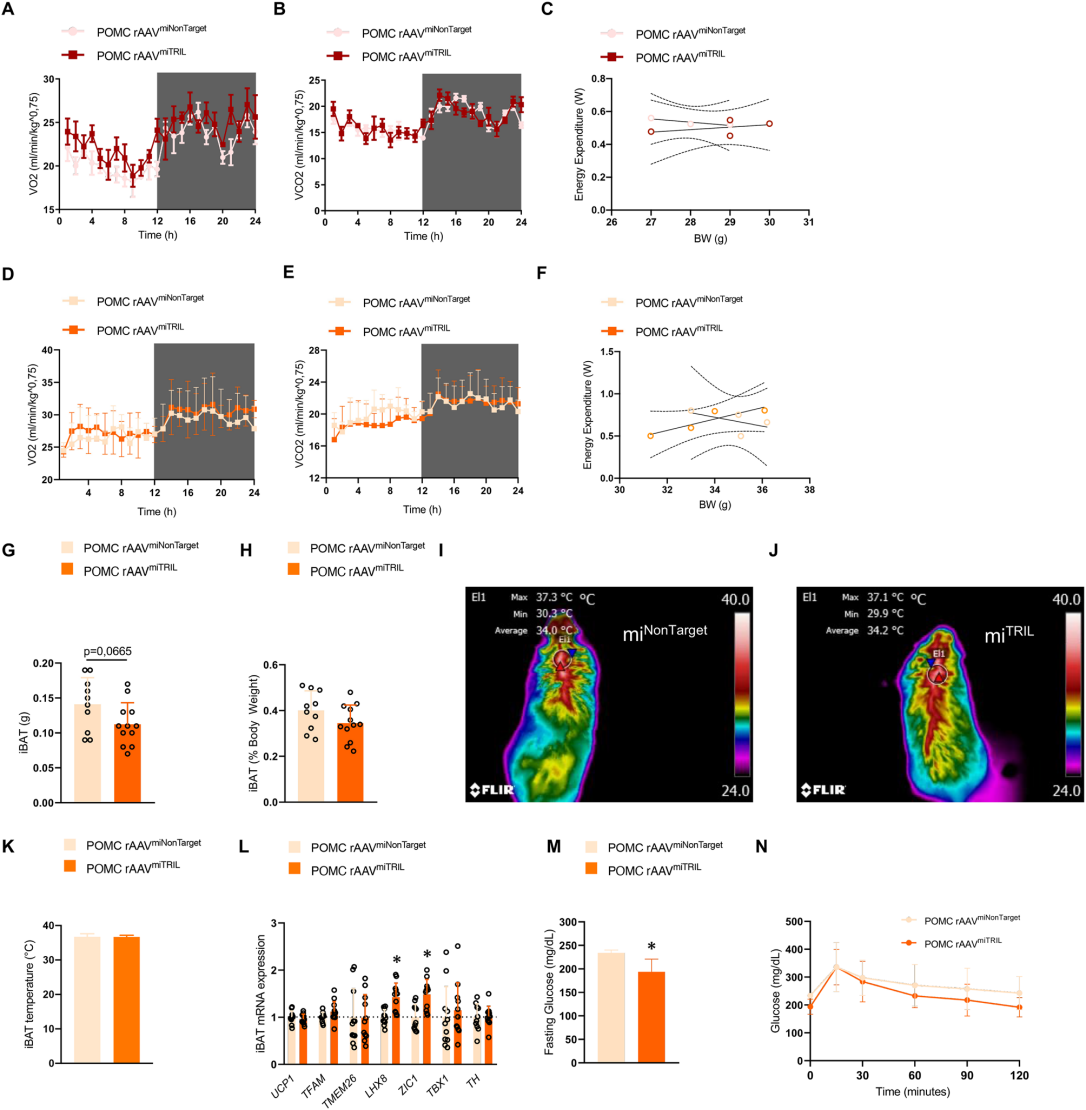
Inhibition of Tril in POMC neurons does not increase energy expenditure. In (**A**–**C**), POMC-Cre mice were submitted to an intracerebroventricular injection with Cre-dependent AAV-FLEX-EGFP-mir30 carrying either a non-target sequence (rAAVmiNonTarget) or a Tril targeting sequence (rAAVmiTRIL) and allowed 2 weeks for recovery, followed by another 2 weeks fed on chow; at the end of the experimental period, mice were submitted to determination of O_2_ consumption (**A**), CO_2_ production (**B**) and energy expenditure (**C**). In (**D**–**N**), POMC-Cre mice were submitted to an intracerebroventricular injection with Cre-dependent AAV-FLEX-EGFP-mir30 carrying either a non-target sequence (rAAVmiNonTarget) or a Tril targeting sequence (rAAVmiTRIL) and then submitted to the same protocol as in Fig. 4C. At the end of the experimental period, mice were submitted to determination of O_2_ consumption (**D**), CO_2_ production (**E**), energy expenditure (**F**), determination of interscapular brown adipose tissue total (**G**) and relative (**H**) mass, determination of interscapular temperature (**I**–**K**) and determination of interscapular brown adipose transcript expression of thermogenic genes (**L**). In addition, blood glucose levels were determined in fasting mice (**M**) and whole-body glucose tolerance was determined by means of an intraperitoneal glucose tolerance test (**N**). In (**A**–**F**), n = 3–4; in (**G**–**N**), n = 12–14. In all, *p < 0.05 vs. miNonTarget. Two-tailed Student’s *t*-test or two-way analysis of variance (ANOVA) followed by Bonferroni post-hoc test were used. The energy expenditure was calculated in watts units (W) using a modified Weir equation and the analysis of covariance (ANCOVA) was performed for the analysis of the effect of body weight on energy expenditure.

No changes in whole-body energy expenditure were observed after POMC-specific knockdown of Tril either in mice fed a chow (Fig. 6A–C) or in mice fed a HFD (Fig. 6D–F). There was a trend to reduce absolute brown adipose tissue (Fig. 6G) but not relative mass (Fig. 6H). Brown adipose tissue temperature was not modified (Fig. 6I–K), but the expression of the thermogenic genes *Lhx8* and *Zic1* were increased in mice under the inhibition of Tril in POMC neurons (Fig. 6L). In addition, the inhibition of Tril in POMC neurons led to lower fasting blood glucose (Fig. 6M) but no change in whole body glucose tolerance (Fig. 6N). In mice fed a chow, the knockdown of Tril in AgRP-expressing neurons did not change food intake (Suppl. Fig. 4A,B), body weight (Suppl. Fig. 4C), VO2 consumption (Suppl. Fig. 4D), VCO2 production (Suppl. Fig. 4E) and energy expenditure (Suppl. Fig. 4F).

### Inhibition of Tril in POMC neurons does not revert the diet-induced obesity phenotype but increases the expression of thermogenic genes in brown adipose tissue

In all preceding experiments, the inhibition of Tril either in the ARC or specifically in POMC neurons occurred two weeks before the introduction of a HFD; thus, the approaches aimed at preventing the harmful effects of the diet. In order to test the hypothesis that the inhibition of Tril in POMC neurons could revert the metabolically adverse effects of long-term consumption of a HFD, mice were fed a HFD for 14 weeks and then injected with the Cre-dependent rAAV^miTril^. After 2 weeks of recovery, mice were maintained for another 8 weeks on HFD and metabolic parameters were evaluated (Suppl. Fig. 2A). As depicted in Suppl. Fig. 2B and C, the inhibition of Tril in POMC neurons could neither revert obesity nor change caloric intake in this group of mice. In addition, there were no changes in epididymal fat mass (Suppl. Fig. 2D,E), brown adipose tissue mass (Suppl. Fig. 2F,G) and brown adipose tissue temperature (Suppl. Fig. 2H–J). Nevertheless, in mice submitted to the inhibition of Tril in POMC neurons, there was a trend to increase brown adipose tissue expression of *Ucp1* and a significant increase in the expression of *Tmem26* (Suppl. Fig. 2K).

### Inhibition of Tril in POMC neurons of obese mice did not change the number of POMC neurons in the arcuate nucleus

**Figure 7:**
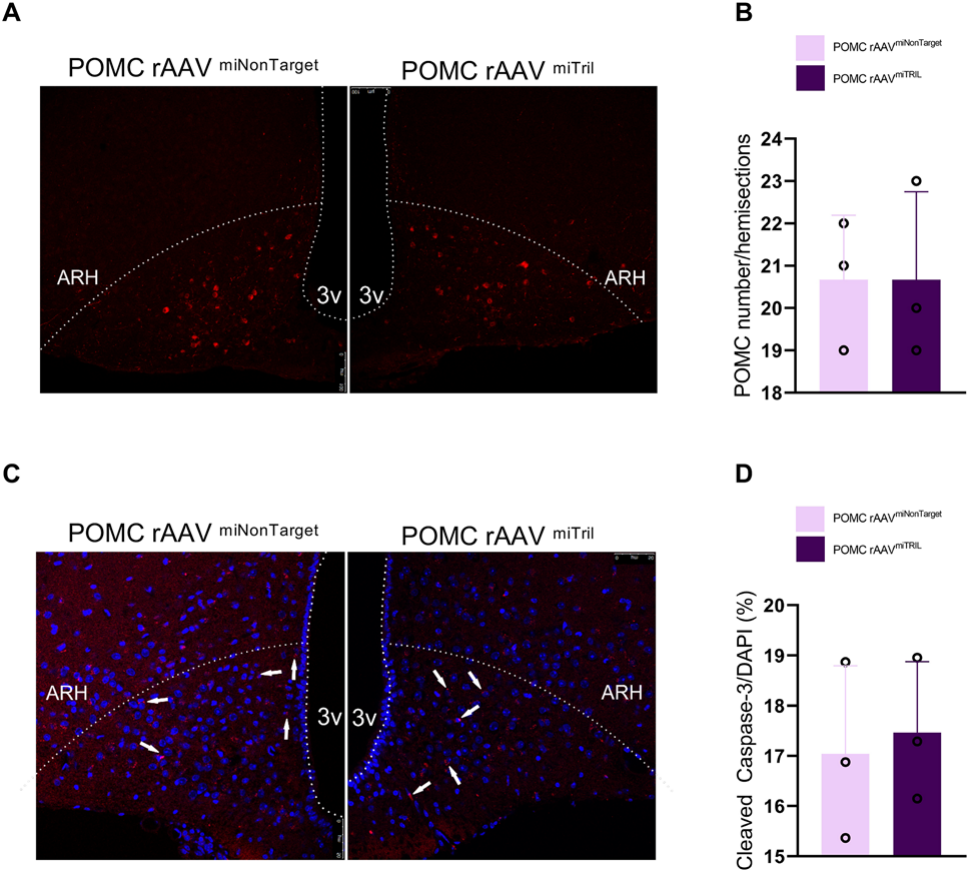
Inhibition of Tril does not prevent POMC loss in obese mice. After 14 weeks fed a HFD mice were assigned to receive either an intracerebroventricular injection of a non-target sequence (rAAVmiNonTarget) or a Tril targeting sequence (rAAVmiTRIL) to inhibit Tril in POMC neurons. Mice were allowed a 2-weeks recovery period and then kept on HFD for 8 weeks (Suppl. Fig. 2A). POMC staining (**A**) and counting (**B**) and Cleaved Caspase-3 staining (**C**) and couting (**D**) were performed after 24 weeks on HFD, as depicted in Suppl. Fig. 2A). In B and D, n = 3. Two-tailed Student’s *t*-test was used.

In experimental obesity, increased apoptosis of neurons in the ARC predominantly affects the number of POMC neurons^9,12,13^. In obese mice submitted to the protocol of inhibition of Tril in POMC neurons (Suppl. Fig. 2A), there were neither changes in the number of POMC neurons (Fig. 7A,B) nor in the cellular expression of cleaved caspase-3 (Fig. 7C,D).

## Discussion

The stability of body mass depends on the functional and structural fitness of ARC neurons, as demonstrated by a number of different experimental approaches^14–16^. Many advancements have been made towards the molecular programs involved in the obesity-associated hypothalamic inflammation and how the neuronal control of energy balance is affected during the course of HFD feeding^3,17,18^.

In DIO, POMC neurons are affected at the functional and structural levels by distinct but integrated mechanisms^9,19–21^. Inflammatory signaling pathways are largely associated with DIO-associated POMC abnormalities^22–24^, and its drivers include the activation of RNA stress granules^25^, endoplasmic reticulum stress^3,26^, PKCt^27^ and TLR4^3,4^. The long-term persistence of hypothalamic inflammation, as it occurs when mice are fed a HFD for longer than 8 weeks, leads to a progressive and potentially irreversible deterioration of the melanocortin system^28,29^. Interestingly, despite the fact that POMC and AgRP neurons share the same anatomical localization, and thus, are potentially exposed to similar harmful factors, POMC undergoes more dramatic changes in response to DIO^9,21^, raising the possibility that POMC-specific factors could be involved in this increased sensitivity to HFD.

Using a POMC-specific transcriptomics^8^, we identified Tril, a TLR4-associated membrane protein previously shown to mediate the inflammatory response to LPS in the brain^7^. Our analysis of in situ mRNA expression revealed that Tril is present in 44% of POMC-expressing neurons and 25% of AgRP-expressing neurons in the ARC. We also employed POMC and AgRP reporter mice to anatomically define the expression of Tril in the hypothalamus and confirmed that Tril is mostly expressed in POMC neurons^8^. In addition, as it could be expected for a TLR4-associated protein, Tril was detected in microglia^30^. It has been recently shown that dietary excess facilitates the infiltration of bone-marrow-derived myeloid cells into the ARC and the activation of resident microglia, leading to an inflammatory responsiveness and neuronal and metabolic dysfunction^31,32^. Using two distinct mouse strains, we showed that hypothalamic Tril mRNA expression is regulated by the consumption of a HFD, as other inflammatory proteins involved in the development of hypothalamic abnormalities in DIO^33–36^.

In order to explore the potential involvement of hypothalamic Tril in the development of HFD-associated obese and metabolic phenotypes, we used two distinct approaches. First, we employed a shRNA-based lentiviral clone to selectively knockdown Tril in a non-cell-specific fashion in the arcuate nucleus. Second, we employed Cre-dependent AAV to specifically inhibit Tril in POMC-expressing neurons. When either inhibition was performed in mice fed chow, there were virtually no changes in the phenotype. Conversely, when either inhibition was performed in mice fed a HFD, there were considerable beneficial changes in the obese and metabolic phenotypes. The different outcomes obtained in mice fed the distinct diets support the potential involvement of Tril in the diet-induced inflammatory response.

The action of Tril is similar to CD14 (serving as an accessory molecule that facilitates the binding of LPS to TLR4), mediating at least part of the inflammatory response triggered in this context^7^. Studies have previously shown that Tril is expressed in glial cells^6,7^. However, as first shown by Henry and coworkers using RNA-seq of hypothalamic neurons^8^ and now, confirmed in this study, Tril is also expressed in POMC neurons. One important aspect of TLR4-associated protein expression in the hypothalamus is that, whether CD14 is expressed in glial cells, it is not expressed in POMC neurons^37^; thus, in this particular cell population, Tril may be essential for the neuronal activation of TLR4.

The non-specific (lentiviral clones) and POMC-specific (Cre-dependent AAV) Tril inhibition in the ARC generated similar but not completely overlapping phenotypes. In both approaches, we found reduction of body adiposity and reduction of BAT mass. In addition, there was a significant reduction of body mass gain after non-specific Tril inhibition and trend for reduction in POMC-specific Tril inhibition. As Tril is also expressed in glial cells^7,30^ and possibly in other ARC neuronal subtypes, some of the effects obtained in the non-specific Tril inhibition might be related to the diet-induced activation of this protein in metabolism-controlling cells other than POMC neurons. For example, GABAergic/non-AgRP neurons were recently identified as the main leptin-responsive, appetite-suppressant subset of ARC neurons^38^. A transcriptomic study revealed that short-term HFD activates hunger-promoting prepronociceptin (PNOC) neurons in the ARC and induces transient overfeeding^39^. Classical neurons, such as those expressing kisspeptin, are involved in the circadian feeding behavior, locomotor activity and thermoregulation^40^.

Previous reports have shown that the inhibition of distinct components of the HFD-induced inflammatory machinery specifically in POMC neurons, such as Myd88, IKK/NFkB, HIF and TGF-βR^2,5,25,41^, resulted in beneficial changes in the metabolic phenotype placing POMC as a direct target for the harmful effects of a HFD. Although recent data have shown that leptin signaling in POMC neurons is dispensable for body weight control^42,43^, the deletion of leptin receptor (LepR) in POMC neurons leads to defective sympathetic innervation of adipose tissue and glucose homeostasis^44^. Here, we showed that inhibition of Tril resulted in a trend to increase STAT3 phosphorylation, suggesting that preserved leptin signaling in POMC neurons might have a role in the improved fasting glucose and increased expression of thermogenic genes in iBAT of mice fed a HFD.

In the final part of the study, we asked if the POMC-specific inhibition of Tril would revert the detrimental effects of long-term DIO. For that, mice were fed a HFD for 14 weeks before the inhibition of Tril in POMC neurons was undertaken. As previously shown, the metabolic outcomes of feeding mice a HFD for shorter than 8 weeks can be reverted by returning mice to chow; however, longer periods on a HFD lead to irreversible metabolic outcomes^28^. In concert with human obesity, studies show that the longer patients remain obese, the more severe the clinical outcomes and the more resilient the obese phenotype is, irrespective of the therapeutic approach employed^45,46^. Here, the inhibition of Tril in POMC neurons in long-term obese mice resulted in no change in body mass, adiposity and food intake. There was only a trend to increase *Ucp1* and a significant increase in *Tmem26* in BAT, suggesting that, despite the lack of effect on the obese phenotype, the inhibition of Tril in long-term obese mice may retain its thermogenic-inducing effect. In addition, the inhibition of Tril in POMC neurons in long-term obese mice was insufficient neither to change the number of arcuate nucleus POMC neurons nor the expression of cleaved-caspase 3. In conclusion, we found that Tril is a component of the HFD-associated hypothalamic dysfunction and its inhibition may partially restore the central control of peripheral metabolism. Pharmacological approaches aimed at inhibiting Tril in the hypothalamus could provide advance in the treatment of obesity.

## Limitations of study

The main limitation of our study is the lack of total and precise deletion of Tril in POMC neurons and some of the marginal effects on metabolic parameters may have a relationship with residual expression of Tril after the knockdown. This gap could be bridged by either crossing a Cre-line with a Tril-floxed mice or employing a selective deletion of Tril in POMC neurons using CRISPR-Cas9.

## Methods

### Animal models

Six-week-old, male, Swiss and C57BL/6J mice were obtained from the University of Campinas experimental animal breeding facility. AgRP-IRES-Cre mice (#012899, Agrptm1(cre)Lowl/J, Jackson Laboratories) were crossed with the Cre-inducible tdTomato reporter mouse (#007909,B6;129S6-Gt(ROSA)26Sortm9(CAG-tdTomato)Hze/J, Jackson Laboratories), and POMC-Cre (#005965, Jackson Laboratories) were used in Cre-dependent rAAV experiments or crossed with Cre-inducible GFP-reporter mice (#004178, The Jackson Laboratory) to determine the colocalization of Tril with fluorescently labeled AgRP and POMC neurons, respectively. Mice were housed individually at 22 °C (± 1 °C) using a 12 h light/12 h dark cycle. All mice had ad libitum access to chow (3.7 kcal g^−1^) or a HFD (60% kcal from fat, 5.1 kcal g^−1^) and water. For refeeding experiments, mice were deprived of the respective diets for 12 h during the dark cycle and thereafter the hypothalamus was harvested 1, 3, 6, 12 and 24 h after refeeding; these experiments were performed after a five-day washout period to eliminate the novelty and the initial overfeeding associated with HFD introduction. All experiments were performed in compliance with current Brazilian legislation, approved by the Ethics Committee of University of Campinas (#4069-1 and #4985-1) and are consistent with the policies and regulations for animal experimentation described in the ARRIVE guidelines.

### Respirometry

To determine O_2_ consumption, CO_2_ production and energy expenditure, mice were acclimatized for 48 h in an open circuit calorimeter system, the LE 405 Gas Analyzer (Panlab—Harvard Apparatus, Holliston, MA, USA). Thereafter, data were recorded for 24 h. Results are presented as the average of light and dark cycles. Analysis of covariance (ANCOVA) was used for the comparison of energy expenditure and body mass in mice.

### Glucose and insulin tolerance tests

Following 6 h of fasting, mice received intraperitoneal (ip) injections with solutions containing glucose (2.0 g/kg body weight) or insulin (1.5 IU/kg body weight) and then blood samples were collected for ip-glucose tolerance test (ipGTT) or ip-insulin tolerance test (ipITT), respectively. Glucose concentrations were measured in tail blood using a portable glucose meter (Optium Xceed, Abbott) at 0, 15, 30, 60 and 120 min after glucose administration or 0, 5, 10, 15, 20, 25 and 30 min after insulin administration.

### Assessment of body composition

To determine total body fat and lean mass, time-domain nuclear magnetic resonance (TD-NMR) was applied using the LF50 body composition mice analyzer (Bruker, Germany). Measurements were performed on the last day of the experiment.

### Thermal images

The estimated iBAT temperatures were determined using an infrared (IR) camera (FLIR T450sc, FLIR systems, Inc. Wilsonville, USA) and analyzed with FLIR-Tools software.

### Tissue collection and histology

C57BL/6J, AgRP tdTomato and POMC-GFP mice were deeply anesthetized with ketamine (100 mg/kg) and xylazine (10 mg/kg) and submitted to transcardiac perfusion with 0.9% saline followed by 4% paraformaldehyde (PFA). Brains were removed, postfixed 24 h in 4% PFA solution and then transferred to a solution containing 20% sucrose in 0.1 M PBS (pH 7.4) for 12 h. Perfused brains were frozen at − 30 °C and sectioned on a cryostat at a thickness of 30 µm. For immunohistochemistry, free-floating sections were washed three times for 10 min with 0.1 M PBS. Next, sections were blocked in 5% donkey serum (Jackson ImmunoResearch, #017-000-121) and 0.2% Triton X-100 in 0.1 M PBS for 1 h at room temperature, followed by incubation in goat anti-TRIL primary antibody (sc-244489, Santa Cruz Biotechnology, Santa Cruz, CA, USA), rabbit anti-IBA1 (Wako Chemicals, #019-19741), rabbit anti-cleaved caspase-3 (Cell Signaling, #9661S), rabbit anti-POMC (Phoenix Pharmaceuticals, #H-029-30), sheep anti-αMSH (Chemicon, #AB5087) and rabbit anti-AgRP (Phoenix Pharmaceuticals, #H-003-53) for 24 h. The sections were then washed three times in 0.1 M PBS and incubated for 2 h at room temperature in donkey anti-goat AlexaFluor^546^ (1:500, Invitrogen, #A-11056), donkey anti-goat FITC (1:500, Santa Cruz, sc-2025), donkey anti-rabbit FITC (1:500, Abcam, ab6798), donkey anti-rabbit IgG AlexaFluor^594^ (1:500, Jackson Immuno Research, #711-585-152) and donkey anti-sheep AlexaFluor^488^ (1:500, Jackson Immuno Research, #713-545-003) conjugated secondary antibodies. Thereafter, the sections were mounted onto slides, and the nuclei were labeled with TOPRO (Life Technologies, T3605). The sections were analyzed with a LEICA TCS SP5 II confocal laser-scanning microscope (Leica Microsystems, Wetzlar, Germany).

### Multiplex fluorescent in situ hybridization (FISH)

Brains from C57BL/6J mice were quickly harvested and frozen in OCT at − 20 °C and stored at − 80 °C. The slides containing a 15-µm-thick brain sections were processed for multiplex FISH using the RNAscope system (ACDBio) and probes for the following mRNA: Tril (C-1), Pomc (C-2) and AgRP (C-3). The pretreatment was performed by sequential incubations of slides in 4% PFA, 50% EtOH, 70% EtOH and 100% EtOH at room temperature. Probe hybridization was achieved by incubation of 30 µl mRNA target probes for 2 h at 40 °C using a HyBez oven. The signal was then amplified by subsequent incubation of Amp-1, Amp-2 and Amp-3 for 30, 30 and 15 min, respectively, at 40 °C using a HyBez oven. Each incubation step was followed by RNAscope washing buffer washes. The slides were coverslipped and mounted using DAPI Fluoromount-G (SouthernBiotech). Slides were visualized with an inverted Zeiss LSM 780 laser scanning confocal microscope using a × 20 lens.

### pSTAT3 staining

Mice were submitted to 12 h of fasting and then received ip injections with mouse leptin (5 mg/kg body weight, Calbiochem, Billerica, MA, USA). After 1 h, mice were submitted to transcardiac perfusion with 0.9% saline followed by 4% PFA. After sectioning on a microtome, the brain sections were rinsed in 0.02 M KPBS (pH 7.4), followed by pretreatment in a water solution containing 1% hydrogen peroxide and 1% sodium hydroxide for 20 min. After extensive washings in 0.02 M KPBS, the sections were incubated in 0.3% glycine for 10 min and then 0.03% lauryl sulfate for 10 min. Thereafter, the sections were blocked in 3% normal donkey serum for 1 h, followed by incubation in rabbit anti-pSTAT3^Tyr705^ (1:1000, Cell Signaling, #91315) for 48 h. For the immunofluorescence reactions, sections were rinsed in KPBS and incubated for 120 min in Fab fragment donkey anti-rabbit AlexaFluor^488^ (1:500, Jackson Immuno Research, #711-547-003). Thereafter, sections were washed three times in 5% formalin for 10 min and then washed three times in 0.02 M KPBS for 5 min. Sections were then incubated with POMC antibody (1:1000. Phoenix Pharmaceuticals, #H-029-30) overnight at room temperature. After washing in 0.1 M PBS, the sections were incubated for 30 min with the secondary antibody AlexaFluor^594^ (1:500, Jackson Immuno Research, #711-585-152) diluted in 0.02 M KPBS. After three washes in 0.02 M KPBS for 10 min, the sections were mounted onto gelatin-coated slides and coverslipped with Fluoromount G (Electron Microscopic Sciences, Hatfield, PA, USA). The percentage of pSTAT3-positive POMC neurons was determined in blind counting by three distinct researchers using hemisections of the middle ARC from four animals for statistical comparison. The sections were processed simultaneously under identical conditions and analyzed with the same microscope set-up.

### Liver histological analysis

Fragments of left lobe were collected and fixed in 10% buffered formalin, embedded in paraffin and stained with hematoxylin and eosin (H&E, Merck, USA), and analyzed and photographed using a light microscope (Axio Observer D1, Zeiss, USA).

### Lentiviral clones

Three different shRNA-based U6-driven lentiviral clones targeting Tril—TRCN0000191275 target sequence: CCAGTATCTTACTGTGTTATT; TRCN0000201889 target sequence: CTGGATTACCTGGATGACCAA; TRCN0000202144 target sequence: CCAACATCTCCTATGCACCAA (Sigma-Aldrich, St Louis, MO, USA) and one scramble (non-targeting) lentiviral particles were used for the overall knockdown experiments. For arcuate nucleus (Arc) bilateral lentiviral delivery, 8–12 week old male C57BL/6J mice were anesthetized with ketamine (100 mg/kg body weight) and xylazine (10 mg/kg body weight) intraperitoneally, and the stereotaxic surgery was carried out using a stereotaxic frame (Stoelting Apparatus, Wood Dale, IL, USA) set at AP − 1.7 mm, ML ± 0.3 mm and DV − 5.6 mm coordinates from Bregma.

### Cre-dependent recombinant adeno-associated viral (rAAV) vectors

With regard to the POMC-specific knockdown of Tril, referred to here as POMC rAAV^miTRIL^, a TRIL-based miRNA construct was constructed by modifying the Cre-dependent AAV-FLEX-EGFP-mir30 (Scn9a) driven by CAG promoter (Addgene plasmid # 79672). Briefly, the plasmid was modified by replacing the self-complementary sequences of Tril shRNA, using EcoRI and Xhol. All miR sequences were preserved. The sequence used was the following, where complementary sequences are underlined: 5′CGAGGCAGTAGGCACCAGTATCTTACTGTGTTATTTACA TCTGTGGCTTCACTAAATAACACAGTAAGATACTGGCGCTCACTGTCAACAGCAATATACCTT-3′. The rAAV particles were produced in the LNBio (Brazilian Biosciences National Laboratory) facility and titrated using an Addgene protocol by qPCR. Briefly, purified AAV particles were treated with DNAse by incubation for 30 min at 37 °C. A standard curve was generated using the AAV-FLEX-EGFP-mir30 (TRIL) plasmid serial diluted. SYBR Green quantitative PCR (qPCR) was performed using the primers: fwd ITR primer 5′-GGAACCCCTAGTGATGGAGTT-3′ and rev ITR primer 5′-CGGCCTCAGTGAGCGA-3′. The Cre-dependent bilateral injections of the rAAV vectors in the Arc were performed in POMC-Cre mice at the following stereotaxic coordinates: AP − 1.7 mm, ML ± 0.3 mm and DV − 5.6 mm from Bregma. All AAVs were allowed 2 weeks for expression before experiments were initiated.

### Gene expression analysis

Total RNA was extracted from the hypothalamus, inguinal and epididymal white adipose tissue, brown adipose tissue and liver using TRIzol reagent (Invitrogen). cDNA synthesis was performed using 2 µg of total RNA. The PCR containing 25 ng of reverse-transcribed RNA was performed using the ABI Prism 7500 sequence detection system (Applied Biosystems). For RT-PCR calculation, the delta CT was used, and the relative gene expression was normalized to that of GAPDH in all samples. In Fig. 1E,F and G the mRNA expression of *Tril* in mice fed on HFD was also normalized for the respective control group of mice fed on chow. The primers used were *Tril* (Mm01330899_s1), *Ucp1* (Mm01244861_m1), *PGC1α* (Mm01188700_m1), *Zic1* (Mm00656094_m1), *LHX8* (Mm00802919_m1), *Tfam* (Mm00447485_m1), *PPARγ* (Mm01184322_m1), *Scd1* (Mm00772290_m1), *Srebf1* (Mm01138344_m1), *Cd36* (Mm01135198_m1, *Tnfα* (Mm00443258_m1), *F4/80* (Mm00802529_m1), *Il1-β* (Mm00434228_m1), *Nlrp3* (Mm00840904_m1), *Hsp90* (Mm00441926_m1), *Hspa5* (Mm00517691_m1), *Caspase*-*3* (Mm01195085_m1), *Pomc* (Mm00435874_m1), *AgRP* (Mm00475829_g1), *Mch* (Mm01242886_g1), *Tmem26* (Mm01173641_m1), *Th* (Mm00447557_m1), *Ccl2* (Mm99999056_m1) and *Tbx1* (Mm00448949_m1).

### Western blotting

Hypothalamic specimens were homogenized in solubilization buffer (1% Triton X-100, 100 mM Tris (pH 7.4), 100 mM sodium 22 pyrophosphate, 100 mM 4 sodium fluoride, 10 mM EDTA, 10 mM sodium vanadate, 2 mM 23 PMSF and 0.1 mg/mL 5 aprotinin). A total of 100 µg of protein per sample were separated by sodium dodecyl sulfate–polyacrylamide gel electrophoresis (SDS-PAGE), transferred to nitrocellulose membranes and blocked in 3% BSA solution in TBST for 2 h. After a washing step, the membranes were blotted with goat-anti TRIL antibody (sc-24489, Santa Cruz Biotechnology, Santa Cruz, CA, USA), and α-tubulin (Sigma-Aldrich, T5168) was used as loading control. Specific bands were labeled by chemiluminescence and were quantified by optical densitometry after exposure to Image Quant LAS4000 (GE Healthcare, Life Sciences).

### Statistical analysis

Results are presented as mean ± standard error of the mean (SEM). Statistical comparisons between different times of refeeding and controls were performed using analysis of variance (ANOVA), followed by Tukey’s post hoc test. Student’s t tests were applied for comparisons between scramble and KD^TRIL^ in experiments with lentiviral clones or POMC Cre^miNonTarget^ and POMC Cre^miTRIL^ in experiments with Cre-dependent rAAV.

## Supplementary Information

Supplementary Information.
