# Supplementary material for: TLR4-interactor with leucine-rich repeats (TRIL) is involved in diet-induced hypothalamic inflammation
Source: Sci Rep. 2021 Sep 9;11:18015. doi: 10.1038/s41598-021-97291-7 (PMC8429592; doi:10.1038/s41598-021-97291-7)
Supplement: Supplementary file 1 — Supplementary Information. [file 41598_2021_97291_MOESM1_ESM.pdf]

Supplementary File

TLR4-interactor with leucine-rich repeats (TRIL) is involved in diet-induced hypothalamic inflammation

Alexandre Moura-Assis[1*], Pedro A.S. Nogueira[1], Jose C. de-Lima-Junior[1], Fernando M. Simabuco[2], Joana M. Gaspar[1], Jose Donato Jr[3], Licio A. Velloso[1,4*]

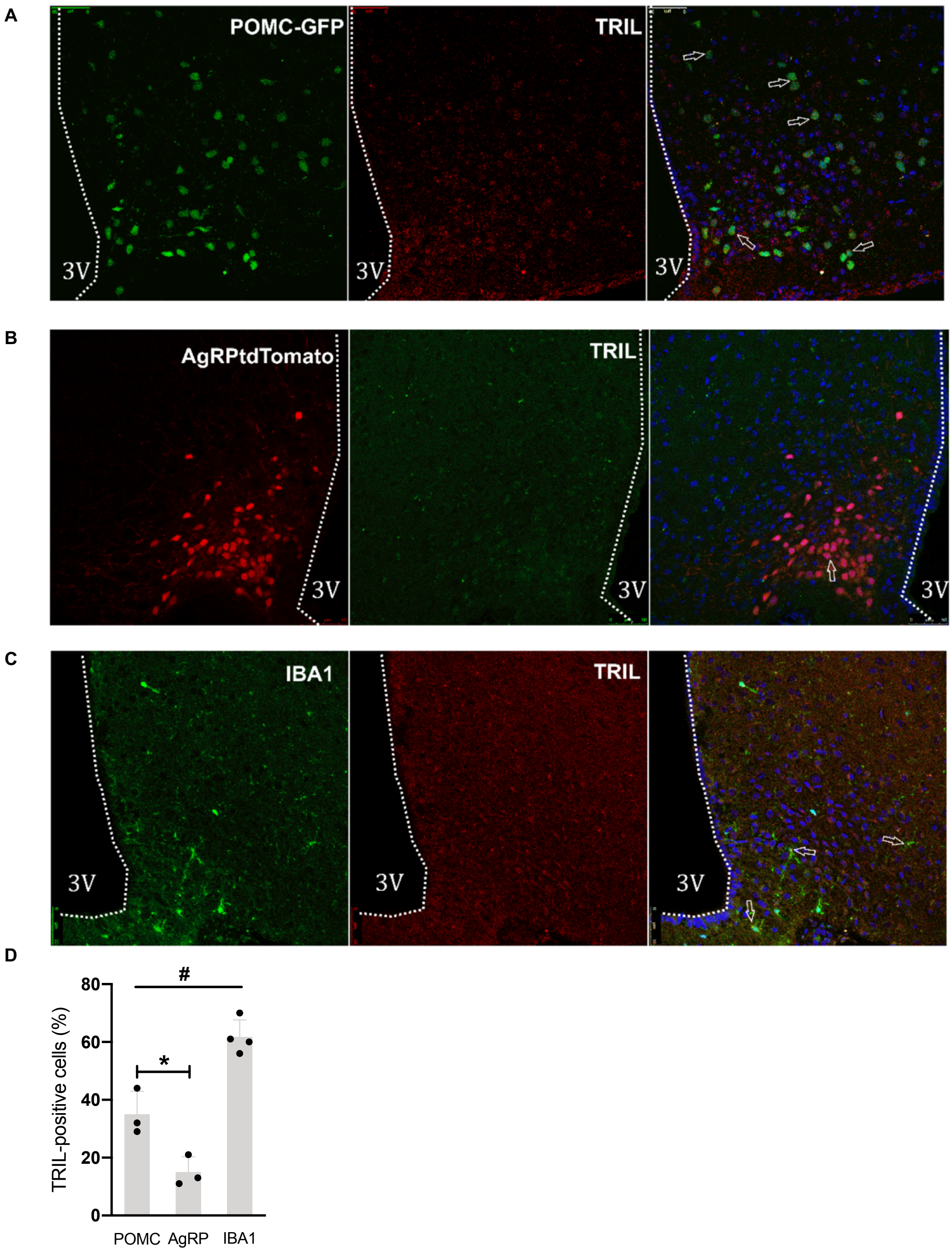

Supplementary Figure 1. Distribution of Tril in POMC-, AgRP-expressing neurons and microglia. Distribution of Tril immunoreactivity in the mediobasal hypothalamus of POMC-GFP mice (Suppl. Fig. 1A), AgRP-tdTomato mice (Suppl. Fig. 1B) and in co-localization with Iba1 in the ARC of wild-type mice fed on chow diet (Suppl. Fig. 1C). Tril-positive cells were quantified (Suppl. Fig. 1D.) 3V, third ventricle.

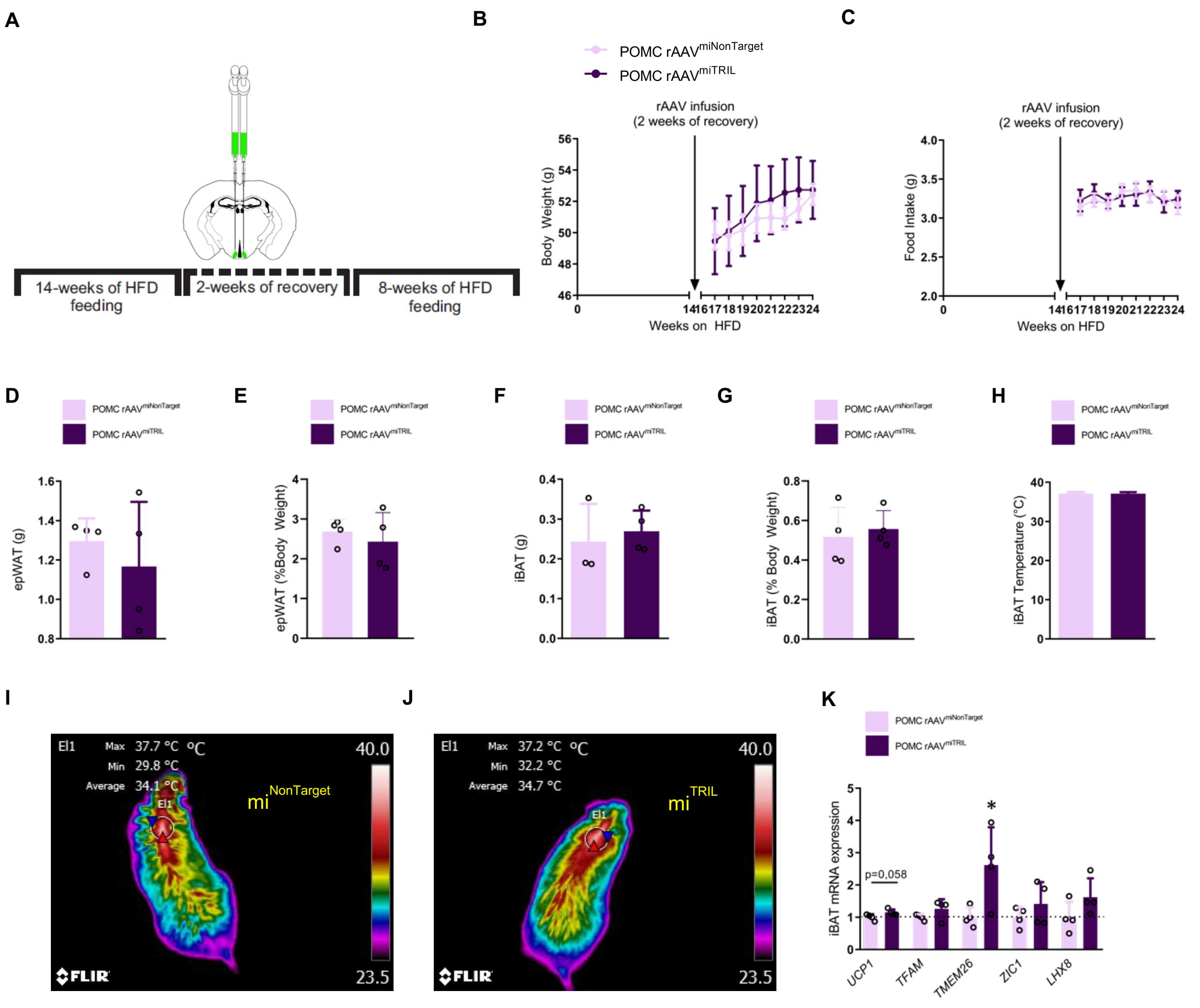

Supplementary Figure 2. Phenotype of obese mice with POMC-specific Tril inhibition. After 14 weeks fed a HFD mice were assigned either to receive an intracerebroventricular injection of a non-target sequence (rAAVmiNonTarget) or a Tril targeting sequence (rAAVmiTRIL) to inhibit Tril in POMC neurons. Mice were allowed a 2-weeks recovery period and then kept on HFD for 8 weeks (Suppl. Fig. 2A). Body weight (Suppl. Fig. 2B) and food intake (Suppl. Fig. 2C) were monitored during the last 8 weeks of experiment. Epididymal WAT mass (Suppl. Fig. 2D), relative epididymal WAT mass (Suppl. Fig. 2E), iBAT mass (Suppl. Fig. 2F) and relative iBAT mass (Suppl. Fig. 2G) were obtained after 24 weeks on HFD as well as the iBAT temperature (Suppl. Fig. 2H, Suppl. Fig. 2I and Suppl. Fig. 2J) and the mRNA expression of thermogenic genes in the iBAT (Suppl. Fig. 2K).

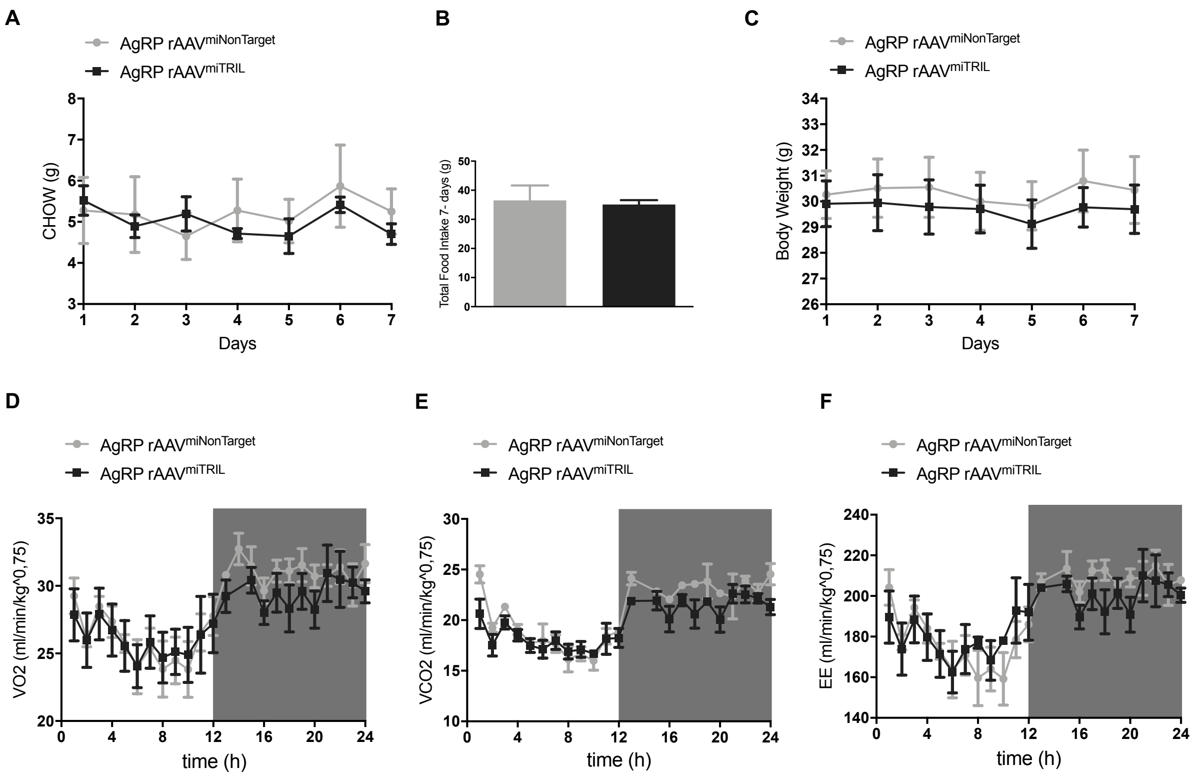

Supplementary Figure 4. Phenotype of mice fed a chow after the knockdown of Tril in AgRP-expressing neurons. AgRP-Cre mice fed a chow were assigned either to receive an intracerebroventricular injection of a non-target sequence ($rAAV^{miNonTarget}$) or a Tril targeting sequence ($rAAV^{miTRIL}$) to inhibit Tril in AgRP neurons. Mice were allowed a 2-weeks recovery period and then kept on chow for 1 week. Food intake (Suppl. Fig. 4A and 4B) and body weight (Suppl. Fig. 4C) were monitored daily. VO2 consumption (Suppl. Fig. 4D), VCO2 production (Suppl. Fig. 4E) and energy expenditure (Suppl. Fig. 4F) were obtained in a 24h trial after 48hours of acclimatation.

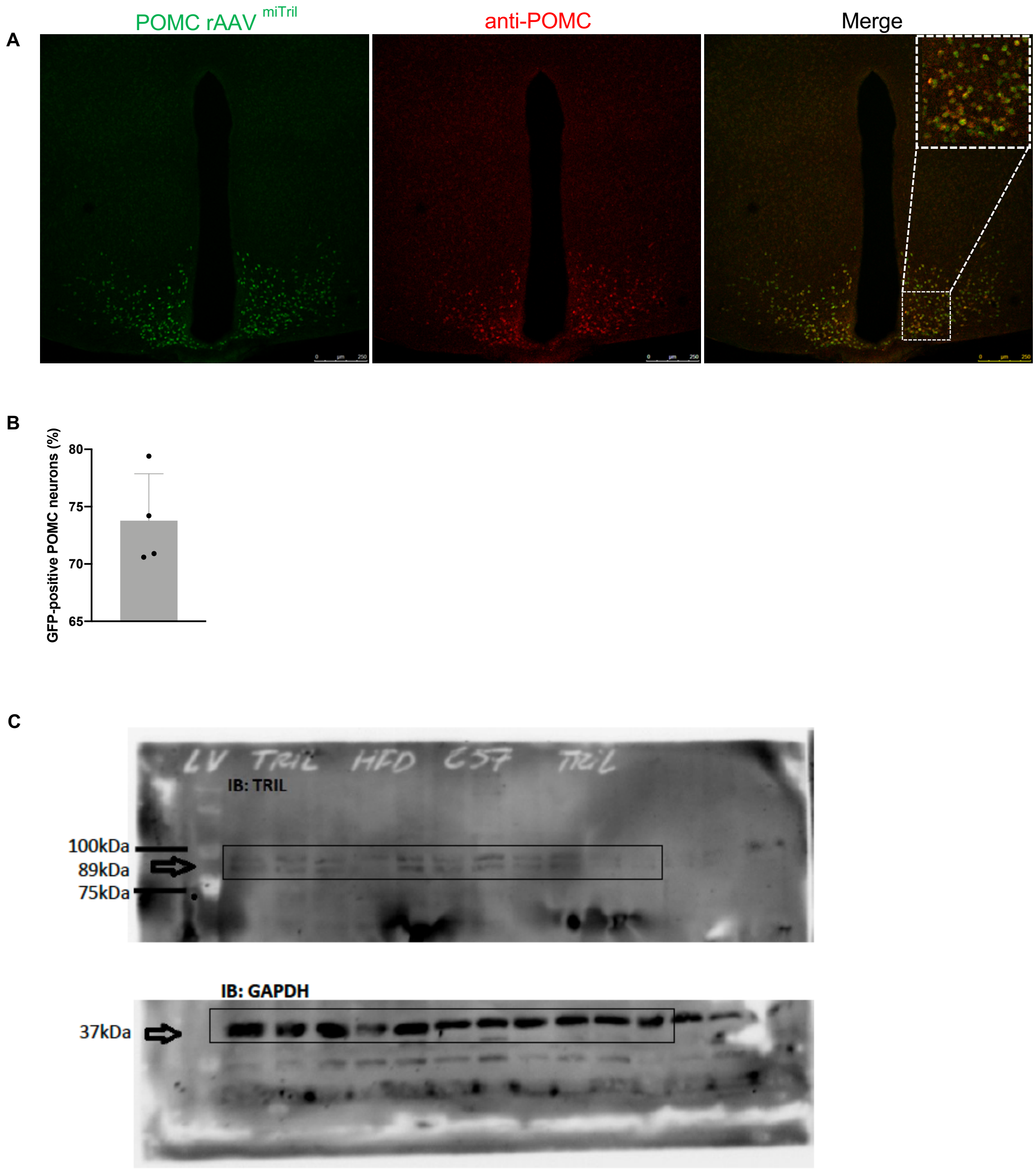

Supplementary Figure 5. Hypothalamic coronal section after bilateral infusion of rAAV^{miTRIL} labeled with GFP in transduced POMC neurons (in red) stained with anti-POMC antibody (Suppl. Fig. 5A). Approximately 74% of POMC neuron were GFP-positive (Suppl. Fig. 5B). Uncropped western blot membrane relative to Figure 2B (Suppl. Fig. 5C).
